# Risk of cardiovascular disease in Chinese patients with rheumatoid arthritis: A cross-sectional study based on hospital medical records in 10 years

**DOI:** 10.1371/journal.pone.0180376

**Published:** 2017-07-05

**Authors:** Kun Zou, Fu-Kun Xiao, Hong-Ying Li, Qiao Zhou, Lu Ban, Min Yang, Chang-Fu Kuo, Weiya Zhang

**Affiliations:** 1 Department of Medical Records and Statistics, Sichuan Academy of Medical Sciences & Sichuan Provincial People’s Hospital, Affiliate Hospital of the University of Electronic Science and Technology, Chengdu, China; 2 Division of Rheumatology, Orthopedics and Dermatology, School of Medicine, University of Nottingham, Nottingham, United Kingdom; 3 Department of Rheumatology and Immunology, Sichuan Academy of Medical Sciences & Sichuan Provincial People’s Hospital, Affiliate Hospital of the University of Electronic Science and Technology, Chengdu, China; 4 Division of Epidemiology & Public Health, School of Medicine, University of Nottingham, Nottingham, United Kingdom; 5 West China Research Center for Rural Health Development, West China School of Public Health, Sichuan University, Chengdu, China; 6 Division of Rheumatology, Allergy and Immunology, Chang Gung Memorial Hospital, Taoyuan, Taiwan; Beijing Key Laboratory of Diabetes Prevention and Research, CHINA

## Abstract

**Objective:**

Though the risk of cardiovascular disease (CVD) in rheumatoid arthritis (RA) has been established in Western population, little is known about the risk in Chinese people with RA. Our objective was to estimate the risk of CVD in Chinese people with RA using hospital medical records data.

**Methods:**

The inpatients medical record database 2005–2015 of Sichuan provincial people’s hospital was examined. All individuals with a primary diagnosis of RA were included as cases, and those of osteoarthritis (OA) were included as controls, which consisted of the unmatched dataset. Then, RA cases and OA controls were matched by sex and age at 1:1 ratio, forming the matched dataset. The morbidity of CVD (including ischemia heart disease (IHD), congestive heart failure (CHF), et al), stroke and arthrosclerosis were extracted from the database, so as the demographic data and comorbidities related to CVD. Multiple logistic regression analysis was used to estimate the risk of CVD in RA adjusted for demographics and comorbidities using the unmatched dataset. Sensitivity analysis was conducted 1) considering interaction terms between RA and comorbidities, and 2) using multivariable conditional logistic regression for the matched dataset.

**Results:**

The unmatched dataset comprised of 1824RA cases and 1995 OA controls and the matched dataset comprised of 1022 pairs of sex and age matched RA and OA patients. RA exhibited increased odds of prevalent CVD compared with OA, and the adjusted ORs (95%CIs) for CVD, stroke, IHD, CHF, and atherosclerosis were1.86(1.42–2.43), 1.11(0.71–1.74), 1.47(0.97–2.24), 2.09(1.03–4.22), and 2.49 (1.97–3.13), respectively, and was 2.26 (1.29–3.96)for IHD further adjusted for interaction term. The matched dataset analysis found similar results.

**Conclusions:**

Chinese people with RA were approximated 2 times more likely to have CVD, IHD, CHF and atherosclerosis compared with those with OA. The findings justified the need of further longitudinal study to establish the causal-relationship between RA and CVD and to estimate the precise risk in this population.

## Introduction

Rheumatoid arthritis (RA) is a chronic, systemic, inflammatory disease associated with persistent inflammatory synovitis, progressive joint destruction, and an excess mortality when compared to the non-RA individuals.[1–5] RA affects estimated 0.42% of the Chinese population, approximately 5,745,600 people.[6] It is evident that RA increases the risk of cardiovascular diseases (CVD) in addition to traditional CVD risk factors.[2] Systemic inflammation and the ‘accelerated atherosclerosis’ in RA are considered as the main mechanism linking RA and CVD.[7] Osteoarthritis (OA)–a leading cause of pain and disability, isgenerally considered as a chronic wear and tear joint condition without systemic inflammation, thus was employed as non-systemic inflammatory comparator in previous studies.[8, 9][4] However, the risk of CVD in Chinese people with RA is unclear and evidence from epidemiological study is lacking. Acknowledge of the relative risk is relevant for optimal management and care of people with RA and prevention of CVD.[10] The objective of this study was to estimate the risk of CVD in Chinese people with RA comparing with those with osteoarthritis (OA) as controls.

## Methods

### Data source and patient definition

The study was approved by the Ethics Committee of Sichuan Provincial People's Hospital (SPPH) on using anonymous medical records data for scientific research purpose (No. 2016–27). This cross-sectional study used the inpatients medical record database (MRD) of SPPH, which records demographics, diagnoses (using ICD-10 codes), procedures (using ICD-CM-9 codes) and expenditure of all inpatients from 2005-2015.All patients with a principal discharge diagnosis of RA (ICD-10 codes: M05.0-M06.9) from 2005–2015 were included, and all patients with a principal discharge diagnosis of OA (ICD-10 codes: M15.0-M19.9) were selected as controls. We focused on the principal diagnosis of discharge to maximize the validity of the case and control definition. Patients with discharge diagnosis of both RA and OA were excluded to avoid the overlapping effect.

Two datasets were generated. The first is an unmatched dataset containing all included RA or OA patients. The second was the matched dataset in which patients with RA and OA were matched at 1:1 ratio by sex and age (per 5 years), which was used for the sensitivity analysis.

### Cardiovascular diseases

The primary outcomes in this study were CVD and stroke. Secondary outcomes were specific cardiovascular conditions: ischaemic heart diseases (IHD), congestive heart failure (CHF) and atherosclerosis (AS). All outcomes were defined by diagnoses recorded in the MRD using ICD-10 codes (S1 Table).[11]

### Covariates or confounders

Confounders considered were age, gender, and comorbidities related to CVD including hypertension, hyperlipidemia, diabetes mellitus or hyperglycaemia (referred as the diabetes mellitus in the rest of the text) and chronic obstructive pulmonary disease (COPD).[9, 12–15]Gender and age were extracted from the MRD. Comorbidities were defined by secondary diagnoses of discharge which were recorded in the database using ICD-10 codes (S1 Table).[11]

### Statistical analysis

Characteristics of participants were described before the association analyses. Continuous and categorical variables were described using mean and standard deviation (SD) and frequency and percentage, respectively. For univariate analysis, ANOVA was conducted for the former, and Chi square test for the latter.

Logistic regression was used to estimate the associations between RA and CVD outcomes using the unmatched dataset, in which unadjusted and adjusted odds ratios (ORs) with its 95% confidence intervals (CIs) were estimated, considering all covariates describe above. Sensitivity analysis was conducted 1) considering significant interaction terms of RA and comorbidities using the unmatched dataset, and 2) using multivariable conditional logistic regression analysis for the matched dataset. Model fit was tested using Bayesian information criterion (BIC).[16] All statistical analyses were conducted using STATA 13.0. The significance level was 0.05.

## Results

### Participants and characteristics

**Fig 1:**
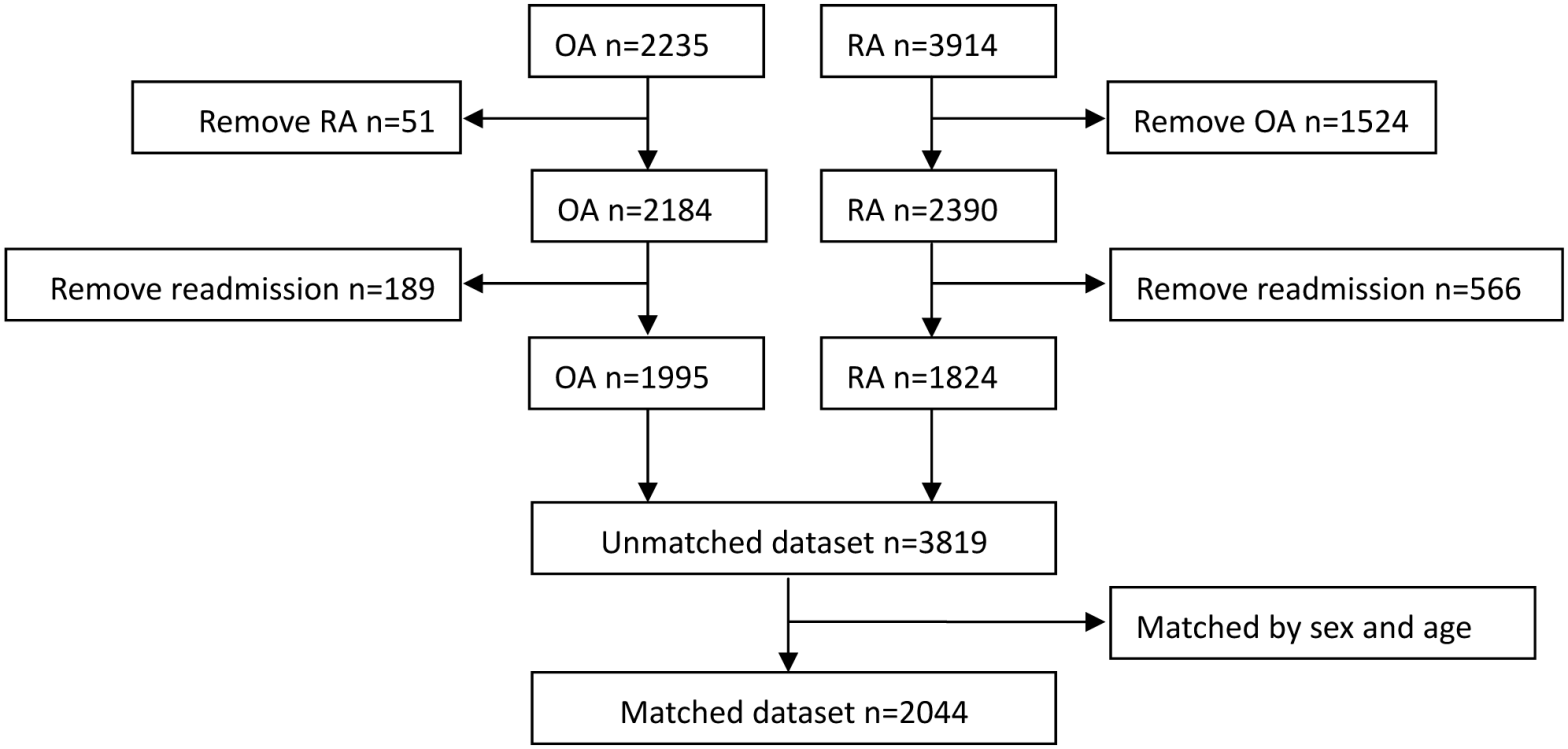
Flow chart of selection of participants.

There were 2,235 OA patients and 3914 RA patients identified from the databasebetween2005 and 2015. After removing patients with discharge diagnoses of both OA and RA, 2184 patients with OA and 2390 with RA remained. Then, readmissions were removed and the last record of a patient hospitalization was selected, leaving 1995 patients with OA and 1824 patients with RA, which consisted of the unmatched dataset of 3819 participants. Finally, RA and OA patients were matched by sex and age (per 5 years), forming the matched dataset of 2044 patients with either OA or RA (1022 each) (Fig 1).

The characteristics of participants with RA (cases) and OA (controls) are presented and compared in Table 1.In the unmatched dataset, patients with OA were significantly older (63.0 vs. 52.4 years), with more women (74.6% vs. 69.6%), hypertension (22.1% vs. 10.6%), hyperlipidemia (11.6% vs. 5.0%) and less COPD (2.0% vs. 4.3%) compared with patients with RA. There was no significant difference of diabetes mellitus between the two groups. In the matched dataset, age, sex, and the proportion of patients with hypertension, hyperlipidemia, IHD or stroke was similar between the two groups. However, there were significantly more diabetes mellitus (p = 0.01) and COPD (p = 0.00) in RA cases than OA controls.

**Table 1:** Characteristics of rheumatoid arthritis (RA) cases and osteoarthritis controls (OA).

	Unmatched	dataset		Matched	dataset	
	OA (%)	RA (%)	p	OA (%)	RA (%)	p
No. of participants	1,995	1,824		1,022	1,022	
Mean age (SD), year	63.0(0.3)	52.4(0.4)	0.00	58.5(0.4)	57.8(0.4)	0.25
Age group, year			0.00			1.00
<45	146(7.3)	553(30.3)		142(13.9)	142(13.9)	
45–49	111(5.6)	191(10.5)		105(10.3)	105(10.3)	
50–54	174(8.7)	202(11.1)		103(10.1)	103(10.1)	
55–59	265(13.3)	243(13.3)		152(14.9)	152(14.9)	
60–64	334(16.7)	229(12.6)		183(17.9)	183(17.9)	
65–69	327(16.4)	178(9.8)		151(14.8)	151(14.8)	
70–74	303(15.2)	114(6.3)		99(9.7)	99(9.7)	
75–79	221(11.1)	66(3.6)		58(5.7)	58(5.7)	
80–84	83(4.2)	37(2.0)		22(2.2)	22(2.2)	
85+	31(1.6)	11(0.6)		7(0.7)	7(0.7)	
Women	1,488(74.6)	1,270(69.6)	0.00	702(68.7)	702(68.7)	1.00
**Comorbidity**						
Hypertension	441(22.1)	194(10.6)	0.00	148(14.5)	167(16.3)	0.24
Hyperlipidemia	231(11.6)	92(5.0)	0.00	75(7.3)	69(6.8)	0.60
Diabetes mellitus	251(12.6)	198(10.9)	0.10	103(10.1)	144(14.1)	0.01
COPD	39(2.0)	78(4.3)	0.00	12(1.2)	64(6.3)	0.00
**Outcomes**						
CVD	166(8.3)	139(7.6)	0.43	53(5.2)	105(10.3)	0.00
IHD	76(3.8)	44(2.4)	0.01	23(2.3)	34(3.3)	0.14
MI	3 (0.15)	2 (0.11)				
CHF	17(0.9)	21(1.2)	0.35	5(0.5)	18(1.8)	0.01
Stroke	74(3.7)	34(1.9)	0.00	29(2.8)	27(2.6)	0.79
Arthrosclerosis	216(10.8)	234(12.8)	0.06	65(6.4)	203(19.9)	0.00

Numbers are frequencies (percentages) unless otherwise specified, CVD: cardiovascular disease, IHD: ischaemic heart disease, MI: myocardial infarction, CHF: congestive heart disease, COPD: chronic obstructive pulmonary disease

### Association analysis

For the unmatched dataset, significant more patients with RA had CVD (adjusted OR: 1.86, 95%CI 1.42–2.43), CHF (adjusted OR: 2.09, 1.03–4.22) and atherosclerosis (adjusted OR: 2.49, 1.97–3.13) than OA patients adjusted for age, sex, hypertension, hyperlipidemia, diabetes mellitus and COPD. However, no difference was found for stroke (adjusted OR: 1.11, 0.71–1.74) and IHD (adjusted OR: 1.47, 0.97–2.24) between the two groups adjusted for other variables. (Table 2)

**Table 2:** Risk of cardiovascular diseases in rheumatoid arthritis (RA) compared with osteoarthritis (OA).

	Unmatched dataset OR (95%CI)		Matched dataset OR (95%CI)	
	Unadjusted	Adjusted	Unadjusted	Adjusted
**Cardiovascular disease**				
RA vs. OA	0.91(0.72–1.15)	1.86(1.42–2.43)*	2.09(1.49–2.95)*	2.19 (1.49–3.21)*
Age, per 5 years	1.42(1.34–1.50)*	1.38(1.30–1.47)*	1.42(1.31–1.54)*	/
Women	0.90(0.69–1.16)	0.94(0.71–1.24)	0.70(0.50–0.98)*	/
Hypertension	4.64(3.63–5.93)*	2.72(2.07–3.57)*	3.79(2.68–5.38)*	1.98(1.07–3.65)*
Hyperlipidemia	2.33(1.68–3.23)*	1.81(1.26–2.58)*	1.67(0.98–2.85)	1.66(0.64–4.32)
Diabetes mellitus	2.74(2.07–3.63)*	1.51(1.11–2.04)*	2.34(1.57–3.49)*	1.57(0.76–3.25)
COPD	2.63(1.62–4.29)*	1.21(0.71–2.05)	3.43(1.93–6.11)*	1.94(0.63–6.01)
**Stroke**				
RA vs. OA	0.49(0.33–0.74)*	1.11(0.71–1.74)	0.93(0.55–1.58)	1.13(0.57–2.25)
Age, per 5 years	1.58(1.43–1.74)*	1.47(1.32–1.64)*	1.54(1.35–1.76)*	/
Women	0.83(0.55–1.26)	0.78(0.50–1.21)	0.75(0.44–1.31)	/
Hypertension	7.38(4.99–10.92)*	3.84(2.50–5.90)*	7.44(4.33–12.79)*	4.80(1.65–13.93)*
Hyperlipidemia	2.72(1.67–4.45)*	1.84(1.08–3.12)*	1.93(0.86–4.34)	1.21(0.26–5.62)
Diabetes mellitus	2.60 (1.66–4.06)*	1.22(0.76–1.96)	2.51(1.35–4.67)*	1.33(0.41–4.25)
COPD	1.91 (0.82–4.44)	0.83(0.34–2.04)	2.05(0.72–5.81)	0.38(0.07–1.93)
**Ischaemic heart disease**				
RA vs. OA	0.62(0.43–0.91)*	1.47(0.97–2.24)	1.49(0.87–2.56)	1.83(0.94–3.56)
Age, per 5 years	1.66(1.51–1.82)*	1.59(1.43–1.76)*	1.74(1.51–2.01)*	/
Women	1.01(0.68–1.52)	1.08(0.70–1.67)	0.78(0.45–1.34)	/
Hypertension	6.49(4.48–9.39)*	3.37(2.25–5.05)*	4.57(2.67–7.83)*	4.40(1.28–15.05)*
Hyperlipidemia	1.83(1.08–3.10)*	1.24(0.70–2.18)	1.58(0.66–3.74)	0.62(0.11–3.64)
Diabetes mellitus	2.61(1.71–3.99)*	1.30(0.83–2.05)	1.99(1.04–3.81)*	1.09(0.35–3.32)*
COPD	2.35(1.12–4.94)*	0.98(0.44–2.17)	2.59(1.01–6.69)*	1.38(0.28–6.87)
**Congestive heart disease**				
RA vs. OA	1.36(0.71–2.58)	2.09(1.03–4.22)*	3.65(1.35–9.86)*	6.95(1.50–32.21)*
Age, per 5 years	1.36(1.18–1.57)*	1.27(1.08–1.49)*	1.29(1.07–1.57)*	/
Women	0.47(0.25–0.90)*	0.57(0.29–1.10)	0.41(0.18–0.94)*	/
Hypertension	4.62(2.43–8.78)*	3.59(1.77–7.29)*	5.18(2.26–11.84)*	13.69(1.13–166.48)*
Hyperlipidemia	0.29(0.04–2.12)	0.22(0.03–1.64)	Exactly the same	(omitted)
Diabetes mellitus	2.72(1.31–5.64)*	1.57(0.73–3.36)	2.04(0.75–5.55)	1.27(0.18–9.18)
COPD	4.96(1.90–12.95)*	2.05(0.72–5.80)	4.00(1.16–13.77)*	3.95(0.19–82.30)
**Atherosclerosis**				
RA vs. OA	1.21 (1.00–1.48)	2.49(1.97–3.13)*	3.65(2.72–4.90)*	4.95(3.36–7.28)*
Age, per 5 years	1.31(1.26–1.37)*	1.31(1.25–1.38)*	1.30(1.22–1.38)*	/
Women	0.92(0.74–1.14)	0.96(0.76–1.21)	0.92(0.70–1.21)	/
Hypertension	3.88(3.14–4.81)*	2.47(1.94–3.15)*	3.34(2.49–4.46)*	1.83(0.98–3.42)
Hyperlipidemia	3.75(2.89–4.88)*	3.51(2.63–4.69)*	3.55(2.43–5.17)*	5.62(2.23–4.14)*
Diabetes mellitus	2.20(1.71–2.84)*	1.21(0.92–1.60)	1.73(1.22–2.44)*	0.91(0.43–1.92)
COPD	3.24(2.14–4.89)*	1.61(1.02–2.54)*	3.05(1.84–5.07)*	1.31(0.35–4.87)

CI: confidence interval, COPD: chronic obstructive pulmonary disease. The number of observations for multivariate conditional logistic regression analysis for cardiovascular disease, ischaemic heart disease, congestive heart disease, stroke and atherosclerosis was 292, 106, 46, 108 and 404 respectively.

*p<0.05

In the sensitivity analysis using matched dataset, the findings were similar to that of unmatched dataset analysis. Significant more patients with RA had CVD (adjusted OR: 2.19, 1.49–3.21), CHF (adjusted OR: 6.95, 1.50–32.21) and atherosclerosis (adjusted OR: 4.95, 3.36–7.28) than OA controls. Still, no difference of patients with stroke (adjusted OR: 1.13, 0.57–2.25) and IHD (adjusted OR: 1.83, 0.94–3.56) was found between the two groups adjusted for other variables.(Table 2)

Moreover, findings were similar for CVD, stroke, CHF and atherosclerosis in the sensitivity analysis considering interaction terms of RA and comorbidities using the unmatched dataset. However, patients with RA were significantly more likely to have IHD (adjusted OR: 2.26, 1.29–3.96) than patients with OA adjusted for other variables and the interaction term of RA and hypertension. (S2 Table)

## Discussion

This study included 3819 Chinese patients with RA or OA consecutively admitted to a medical centre between 2005 and 2015. Important covariables such as age, sex, and comorbidities related to CVD were adjusted in the estimate of association between RA and CVD. RA and OA, CVD, and comorbidities related to CVD were defined by physician’s diagnoses recorded as ICD-10 codes in MRD, which reduced information bias and enhanced the accuracy of variable definition. We found 1) the prevalence of CVD in Chinese patients with RA admitted to hospital was 7.6%-10.3%; 2) RA patients were approximately twice more likely to have CVD, IHD, CHF and atherosclerosis than people with OA; and 3) the risk of stroke were not significantly different between the two groups, while an incremental (from non-significant to significant)risk of IHD was found when more covariables or their interaction were adjusted.

The approximately 2 folded risk of total CVD, IHD and CHF in Chinese people with RA found in this study is comparable to previous studies in other populations (mainly Caucasians).[2, 17, 18] For Chinese population, Chung and colleagues found a38% increase of risk of acute myocardial infarction in patients with RA comparing with non-RA population in a cohort study in Taiwan; unfortunately the risk of total CVD and other specific CVD were not estimated.[19] The association between RA and CVD is further supported by our finding that the risk of atherosclerosis increased in RA, for it is considered as the main mechanism underlying RA and CVD morbidity linked by systemic inflammation.[7, 20] However, we found a positive but non-significant risk of stroke, and an incremental risk (from non-significant to significant)of IHD in people with RA when more covariables or interaction were adjusted. The increased risk of stroke and IHD in RA has been shown in large sample sized longitudinal studies and meta-analysis in other population.[2, 18] The non-significant finding of stroke and IHD may be due to the small number of events and lack of power in this study. Furthermore, the risk of CVD in RA compared with OA may not be able to be directly interpreted as the risk compared with non-RA general population. Because evidence has emerged recently that OA may also increase the risk of CVD compared with the general population, though it is still controversial.[21, 22] Therefore, the CVD risk in Chinese people with RA comparing with the general population might be higher than the estimate in our study.

This study has several limitations. Firstly, the participants in this study were all inpatients consecutively admitted to a medical centre. Therefore, selection bias may be introduced, for inpatients are generally more severe in disease status than outpatients or community patients, but this situation may be even between RA cases and OA controls. Secondly, some covariables or confounders of CVD were not included in the analysis for they were absence in the database such as obesity, smoking and anti-rheumatic drugs. There is evidence that obesity is more prevalent in patients with OA than in RA.[17, 21] Thus, the risk of CVD in RA compared with OA may be higher if obesity was controlled for. Furthermore, diabetes and COPD were adjusted in the analysis, which are positively associated with obesity and smoking, thus their effect on CVD may be indirectly and partially adjusted. Thirdly, detailed analysis on sub-category of CVDs such as myocardial infarction was not possible due to the small number of events. Finally, the natural of the cross-sectional study design prevented us to yield a causal-relationship between RA and CVD.

In conclusion, Chinese people with RA were approximated 2 times more likely to have CVD, IHD, CHF and atherosclerosis compared with people with OA. Further longitudinal study is needed to establish the risk of total and specific CVD in Chinese people with RA.

## Supporting information

S1 Table ICD-10 codes of outcomes and covariates. (DOCX)

S2 Table Sensitivity analysis using unmatched dataset adjusted for covariables and interaction terms with rheumatoid arthritis (RA). RA: rheumatoid arthritis; OA: osteoarthritis, COPD: chronic obstructive pulmonary disease; ^&^ Diabetes mellitus or hyperglycemia; ^$^ No significant interaction term was detected; ^+^Bayesian information criterion in favor of the model; *p<0.05.
(DOCX)

S1 File Strobe checklist. (DOC)
