# Supplementary material for: Risk of cardiovascular disease in Chinese patients with rheumatoid arthritis: A cross-sectional study based on hospital medical records in 10 years
Source: PLoS One. 2017 Jul 5;12(7):e0180376. doi: 10.1371/journal.pone.0180376 (PMC5498026; doi:10.1371/journal.pone.0180376)
Supplement: S1 Table — (DOCX) [file pone.0180376.s001.docx]

# Supporting information

S1 Table. ICD-10 codes of outcomes and covariates

| Variable | ICD-10 code |
| --- | --- |
| **Outcomes** |  |
| Cardiovascular disease |  |
| Rheumatic heart disease | I05.0-I09.9 |
| Ischemic heart disease | I20.0-I25.9 |
| Myocardial infarction | I21.x, I22.x, I25.2 |
| Pericarditis | I30.0-I32.9 |
| Endocarditis | I33.0-I33.9 |
| Valve disorders | I34.0-I37.9 |
| Myocarditis | I40.0-I41.9 |
| Cardiomyopathy | I42.0-I43.9 |
| Conduction disorders | I44.0-I45.9 |
| Arrhythmias | I46.0-I49.9 |
| Congestive heart failure | I50.0-I50.9 |
| Other heart disease | I51.0-I51.9 |
| Atherosclerosis | I70.0-I70.9 |
| Stroke | I60.0-I64.9 |
|  | H34.1 |
|  | G45.0-G45.9 |
| **Covariable** |  |
| Hypertension | I10.0-I15.9 |
| Hyperlipidemia | E78.0-E78.5 |
| Diabetes mellitus | E10.0-E14.9 |
| Hyperglycaemia | R73.0-R73.9 |
| Chronic obstructive pulmonary diseases | J40.0-J44.9 |
|  | J47.0-J47.9 |
