# Supplementary material for: Risk of cardiovascular disease in Chinese patients with rheumatoid arthritis: A cross-sectional study based on hospital medical records in 10 years
Source: PLoS One. 2017 Jul 5;12(7):e0180376. doi: 10.1371/journal.pone.0180376 (PMC5498026; doi:10.1371/journal.pone.0180376)
Supplement: S2 Table — RA: rheumatoid arthritis; OA: osteoarthritis, COPD: chronic obstructive pulmonary disease; & Diabetes mellitus or hyperglycemia; $ No significant interaction term was detected; +Bayesian information criterion in favor of the model; *p<0.05. (DOCX) [file pone.0180376.s002.docx]

# Supporting information

S2 Table. Sensitivity analysis using unmatched dataset adjusted for covariables and interaction terms with rheumatoid arthritis (RA)

|  | Adjusted OR (95%CI) | | |
| --- | --- | --- | --- |
| **Cardiovascular disease^+^** |  |  |  |
| RA vs. OA | 2.12 | (1.60-2.83)* | |
| Age (per 5 years) | 0.93 | (0.70-1.23) | |
| Women | 1.38 | (1.30-1.47)* | |
| Hypertension | 2.76 | (2.10-3.63)* | |
| Hyperlipidemia | 2.47 | (1.64-3.71)* | |
| Diabetes mellitus^&^ | 1.49 | (1.10-2.02)* | |
| COPD | 1.17 | (0.69-2.00) | |
| RA # Hyperlipidemia | 0.29 | (0.11-0.71)* | |
| **Stroke^$^** |  |  |  |
| RA vs. OA | 1.11 | (0.71-1.74) | |
| Age (per 5 years) | 0.78 | (0.50-1.21) | |
| Women | 1.47 | (1.32-1.64)* | |
| Hypertension | 3.84 | (2.50-5.90)* | |
| Hyperlipidemia | 1.84 | (1.08-3.12)* | |
| Diabetes mellitus^&^ | 1.22 | (0.76-1.96) | |
| COPD | 0.83 | (0.34-2.04) | |
| **Ischemic heart disease** |  |  |  |
| RA vs. OA | 2.26 | (1.29-3.96)* | |
| Age (per 5 years) | 1.08 | (0.70-1.67) | |
| Women | 1.59 | (1.43-1.76)* | |
| Hypertension | 4.84 | (2.87-8.18)* | |
| Hyperlipidemia | 1.23 | (0.70-2.17) | |
| Diabetes mellitus^&^ | 1.25 | (0.79-1.96) | |
| COPD | 1.01 | (0.46-2.23) | |
| RA # Hypertension | 0.37 | (0.16-0.88)* | |
| **Congestive heart failure** |  |  |  |
| RA vs. OA | 2.69 | (1.28-5.65)* | |
| Age (per 5 years) | 0.54 | (0.28-1.04) | |
| Women | 1.28 | (1.09-1.51)* | |
| Hypertension | 3.65 | (1.80-7.42)* | |
| Hyperlipidemia | 0.20 | (0.03-1.54) | |
| Diabetes mellitus^&^ | 1.54 | (0.71-3.30) | |
| COPD | 6.93 | (1.82-26.31)* | |
| RA # COPD | 0.12 | (0.02-0.93)* | |
| **Atherosclerosis** |  |  |  |
| RA vs. OA | 2.60 | (2.05-3.31)* | |
| Age (per 5 years) | 0.95 | (0.75-1.20) | |
| Women | 1.32 | (1.25-1.39)* | |
| Hypertension | 2.47 | (1.94-3.15)* | |
| Hyperlipidemia | 3.50 | (2.62-4.68)* | |
| Diabetes mellitus^&^ | 1.21 | (0.92-1.60) | |
| COPD | 2.73 | (1.32-5.63)* | |
| RA # COPD | 0.44 | (0.17-1.09) | |

RA: rheumatoid arthritis; OA: osteoarthritis, COPD: chronic obstructive pulmonary disease; ^&^ Diabetes mellitus or hyperglycemia; ^$^ No significant interaction term was detected; ^+^Bayesian information criterion in favor of the model; *p<0.05
